# Reduced Wiskott-Aldrich syndrome protein expression in preeclampsia placenta impairs trophoblast syncytialization by modulating syncytin-2 via FAK/β-catenin pathway

**DOI:** 10.3389/fcell.2025.1678878

**Published:** 2025-11-06

**Authors:** Shuo Zhang, Jiao Wang, Yunpeng Ge, Pin Cao, Zekai Bai, Hongfei Shen, Dan Wang, Chong Qiao

**Affiliations:** 1 Department of Obstetrics and Gynecology, Shengjing Hospital of China Medical University, Shenyang, China; 2 Department of Anesthesiology, The First Affiliated Hospital of China Medical University, Shenyang, China; 3 Key Laboratory of Maternal-Fetal Medicine of Liaoning Province, Shenyang, China; 4 Key Laboratory of Obstetrics and Gynecology of Higher Education of Liaoning Province, Shenyang, China

**Keywords:** N-WASP, preeclampsia, trophoblast, syncytin-2, syncytialization

## Abstract

**Objective:**

We investigated the role of neural Wiskott-Aldrich syndrome protein (N-WASP) in preeclampsia (PE), focusing on its regulatory impact on trophoblast syncytialization.

**Methods:**

We analyzed placental samples from patients with PE (n = 30) and controls (n = 35) using RNA extraction, quantitative real-time polymerase chain reaction, Western blot, and immunohistochemistry. BeWo cell lines were used to model trophoblast fusion under forskolin stimulation. We explored N-WASP’s role in trophoblast cell behavior using gene knockdown and overexpression experiments. Using bioinformatics analyses and molecular docking studies, we elucidated the interaction between N-WASP and associated pathways. These findings were validated *in vivo* using an L-NAME PE rat model.

**Results:**

N-WASP expression was significantly reduced in PE placentas, correlating positively with syncytin-2 and GCM1 levels. In BeWo cells, N-WASP promoted syncytialization by activating the FAK/β-catenin pathway, causing increased nuclear β-catenin translocation, glial cells missing 1 expression, and syncytin-2 transcription. Mechanistically, N-WASP interacted with myosin 1B causing FAK pathway activation. Restoring N-WASP expression ameliorated placental abnormalities and PE symptoms *in vivo*. We identified hydroxychloroquine as a potential N-WASP agonist, capable of enhancing trophoblast syncytialization *in vitro* using molecular docking. Treatment with hydroxychloroquine significantly improved clinical symptoms, including reducing elevated blood pressure, decreasing urinary protein levels, and normalizing serum creatinine concentrations in PE rat models.

**Conclusion:**

We identified N-WASP as a key regulator of trophoblast syncytialization through the FAK/β-catenin signaling pathway, influencing syncytin-2 expression. The findings reveal a novel molecular mechanism underlying PE and suggest that N-WASP is a potential therapeutic target for PE.

## 1 Introduction

Preeclampsia (PE) is a severe idiopathic disease that considerably affects pregnancy, posing substantial risks to both maternal and fetal health (Fishel Bartal and Sibai, 2022). The etiology and pathogenesis of PE are intricate and multi-factorial, including a multi-step process that is not fully understood. The placenta contributes substantially to PE development (Roland et al., 2016; Staff, 2019; Melchiorre et al., 2022). Defects in placentation can influence the disease onset, including abnormalities in cytotrophoblast (CTB) cell fusion (Knofler et al., 2019), syncytiotrophoblast (STB) function (Jones and Fox, 1980; Apicella et al., 2019), inadequate trophoblast invasion, and spiral artery remodeling. Furthermore, impaired trophoblast syncytialization and fusion of CTB cells to form the multinucleated STB are obvious events in PE development (Hahn et al., 2022). The STB is essential for maintaining the integrity and functionality of the placental barrier, which facilitates nutrient exchange, hormone production, and immune protection between mother and fetus. In PE, the placental barrier becomes compromised, which contributes to the pathology of the disease when this process is disrupted (Huppertz, 2008; Zheng et al., 2023). Notably, Syncytin-2, a membrane glycoprotein encoded by the human endogenous retrovirus (*HERV*), is crucial at the CTB-STB interface. Particularly, it helps with CTB fusion and has a greater functional significance in this process than syncytin-1 (Roberts et al., 2021). In PE, reduced expression of syncytin-2 is associated with decreased trophoblast fusion and impaired STB formation (Vargas et al., 2011; Chen et al., 2008). The transcription factor glial cells missing 1 (GCM1) regulates syncytin-2 expression and is essential for CTB differentiation into STB (Zhang et al., 2021). However, disruption in GCM1 function is associated with impaired trophoblast syncytialization and PE development (Wang L. J. et al., 2019), hence it is vital in placental development and function.

Additionally, syncytialization is a complex and highly regulated process that involves the rearranging of the actin cytoskeleton (Hernandez and Podbilewicz, 2017). In addition, Neural Wiskott-Aldrich syndrome protein (N-WASP) is an important member of the WASP family encoded by the *WASL* gene. It is a nucleation promoting factor that drives the actin cytoskeleton dynamics (Zheng et al., 2023). N-WASP influences various biological functions, including tissue morphogenesis (Hawkins et al., 2021) and cell migration and invasion (Antón et al., 2020) by controlling cytoskeleton rearrangement. Notably, high concentrations of N-WASP are associated with adverse clinical prognoses and low survival rates in various tumors (Liu et al., 2015; Yan P. et al., 2020; Kurisu and Takenawa, 2010). Moreover, N-WASP is required for myoblast fusion during myogenesis in *Drosophila* (Mukherjee et al., 2011) and specifically contributes to myogenic cell-cell fusion in mice (Gruenbaum-Cohen et al., 2012). However, its role in trophoblast cells remains unknown.

In this study, we revealed for the first time the correlation between N-WASP, syncytin-2, and PE. We found that N-WASP regulates trophoblast fusion by influencing the key molecule syncytin-2 through systematic research on human placental samples, the BeWo cell line, and rat models of PE. Hence, it is important in the pathogenesis of PE. In this study, we provided new insights into the molecular mechanisms of PE and highlighted the potential of N-WASP as a therapeutic target.

## 2 Materials and methods

### 2.1 Patients and placenta samples collection

We enrolled 65 patients who gave birth at Shengjing Hospital of China Medical University between July 2020 and December 2021 in this trial. This research was approved by the Ethics Committee of Shengjing Hospital of China Medical University (license number: 2021PS890K). Of these 65 patients, 30 were preeclamptic, and 35 were normal pregnant women matched by gestational week. PE was diagnosed following the guidelines of the American College of Obstetricians and Gynecologists (Gestational Hypertension and Preeclampsia, 2020). Notably, patients with multiple pregnancy, primary hypertension, diabetes and chronic nephritis, chorioamnionitis and abnormal fetal development were excluded.

### 2.2 RNA extraction and quantitative real-time polymerase chain reaction

We extracted total RNA from placenta tissues and cells using RNAiso plus (9108, TaKaRa, Japan). Additionally, RNA concentration and quality were determined using NanoDrop™ One/OneC MicroUV-vis spectrophotometer (Thermo Fisher Scientific Inc.). Isolated RNA was reverse transcribed to cDNA using PrimeScript^™^ RT Master Mix (Perfect Real Time) (RR036A, TAKARA, Japan). Furthermore, quantitative real-time polymerase chain reaction (RT-qPCR) was performed using the Applied Biosystem 7500 *Fast* instrument employing TB Green® *Premix Ex Taq* ^™^ II (Tli RNaseH Plus) (RR820A, TAKARA, Japan). Analysis was performed using the 7500 Fast System Software. The results were presented using cycle threshold and analyzed using the 2^-△△CT^ method to normalize the relative expression of reference gene Glyceraldehyde-3-Phosphate Dehydrogenase (GAPDH). Each sample was assayed in triplicate wells and tested three times.

The primer sequences are as follows:

*WASL* (Human),

forward: GCTCTGGACGAGATGCACTGTTAG,

reverse: CAGGTGTTGGTGGTGTAGACTCTTG;

*ERVFRD-1* (Human)

forward: ACTAGCAGCCTACCGCCATCC,

reverse: CCCTGGTGTTTCAGTGGAAGAGC;

GAPDH,

forward: CTGGGCTACACTGAGCACC,

reverse: AAGTGGTCGTTGAGGGCAATG;

β-human chorionic gonadotropin (HCG),

forward: AATTGGGTCCGCTGACTCTG,

reverse: TATCTTCCGCAAGCACTGGG.

### 2.3 Western blot

Whole proteins of placenta and cells were extracted using RIPA Lysis Buffer (P0013B, Beyotime, China) combined with Halt Protease Inhibitor Cocktail (87786, Thermo Fisher, United States). The BCA Protein Assay Kit (P0012, Beyotime, China) was used to assay the protein concentration. Equal amounts of protein (30 μg) were loaded for 10% sodium dodecyl sulfate-polyacrylamide gel electrophoresis. The proteins were transferred to the polyvinylidene fluoride membrane (IPVH00010, Millipore, United States) membranes. Subsequently, the membranes were blocked with a 5% non-fat dry milk/TBST buffer for 2 h at room temperature. The primary antibodies were incubated overnight at 4 °C. The membranes were washed using TBST for 10 min repeated three times and incubated in the secondary antibody (1:5000, SA00001-2, Proteintech) for 2 h at room temperature. SuperSignal West Pico PLUS Luminol/Enhancer (34580, Thermo Scientific, United States) was used to expose images through the Automatic chemiluminescence image analysis system (5200, Tanon, China). The concentrations of antibodies were *WASL* Polyclonal antibody (1:2000, 14306-1-AP, Proteintech); Rabbit Anti-*ERVFRD-1* antibody (1:2000, bs-15466R, Bioss), GAPDH Monoclonal antibody (1:3000, 60004-1-Ig, Proteintech); GCM1 Polyclonal antibody, (1:1000, 21724-1-AP, Proteintech); E-cadherin Polyclonal antibody (1:1000, 20874-1-AP, Proteintech); Beta Catenin Polyclonal antibody (1:1000, 51067-2-AP, Proteintech); GSK3β Polyclonal Antibody (1:1000, YT2082, Immunoway); GSK3β(phospho Ser9) Polyclonal Antibody (1:1000, YP0124, Immunoway); Lymphoid Enhancer-Binding Factor (LEF1) Antibody (N-term) (1:1000, AP12048A, Abcepta); β-actin (1:20000, 66009-1-Ig, Proteintech).

### 2.4 Immunohistochemistry

The placenta tissue paraffin sections (5 μm) were dewaxed using xylene and rehydrated with gradient ethanol dilutions. The sections were put into the citrate antigen repair solution and boiled in a microwave oven. Subsequently, they were cooled to room temperature naturally. UltraSensitive™ SP IHC Kit (KIT-9710, MXB Biothchnologies, China) and DAB Kit (DAB-0031, MXB Biothchnologies, China) were used for immunohistochemical staining. The primary antibodies were diluted in phosphate-buffered saline (PBS): *WASL* Polyclonal antibody, 1:100; Rabbit Anti-*ERVFRD-1* antibody, 1:100; GCM1 Polyclonal antibody, 1:50; Cytokeratin 7 Polyclonal antibody (1:100, Proteintech, China); E-cadherin Polyclonal antibody, 1:200. Negative controls were performed with PBS. A positive stain was defined as brownish-yellow color change of tissues under the optical microscope. In addition, hematoxylin was used to stain the nuclei for 5 min. Imagines acquisition and analysis were performed by using NIS-Elements software (Nikon).

IHC Scoring (Immunoreactive Score, IRS): Immunostaining results for all target proteins were semi-quantitatively assessed using the immunoreactive score (IRS) method under blinded conditions. The IRS integrates the percentage of positive cells and staining intensity into a composite score. Each specimen received two subscores: the percentage of positive cells (PP) and staining intensity (SI), defined as follows: PP: 0 = 0%; 1 = ≤25%; 2 = 26–50%; 3 = 51–75%; 4 = >75%. SI: 0 = no staining; 1 = light yellow (weak); 2 = brownish-yellow (moderate); 3 = brown (strong). The final IRS was calculated as PP × SI, ranging from 0 to 12. All slides were independently evaluated by five blinded observers to ensure scoring objectivity.

### 2.5 Cell culture

We purchased the BeWo (Human chorionic tumor cells) cells from Wuhan Pricella Biotechnology Co., Ltd. (CL-0500, Procell, China). These cells were maintained in Ham’s F-12K (PM150910, Procell, China) with 10% fetal bovine serum (164210, Procell, China) and 1% Penicillin-Streptomycin (P/S). The cell was maintained in a humidified atmosphere of 5% carbon dioxide at 37 °C. Subsequently, the cell fusion was induced by Forskolin (F3917, Sigma-Aldrich, United States). 5 × 10^^5^ BeWo cells were seeded in a six-well plate, and after 48 h, when the cells had adhered firmly, Forskolin was added for 48 h to induce fusion. The concentration gradients used in the dose-response experiment were 0 μM, 10 μM, 25 μM, and 50 μM. For subsequent experiments, a concentration of 25 μM was used for treatment.

### 2.6 Cell fusion index

We calculated the cell fusion index using the immunofluorescence microscopy images of E-cadherin staining. From each coverslip, five randomly selected, non-overlapping areas were assessed for cell fusion. The index was determined using the formula (N-S)/T (where N is the number of nuclei in syncytia, S is the number of syncytia, and T is the total number of nuclei) (Lin et al., 2011). This measurement was replicated in at least three independent experiments.

### 2.7 shRNA transfection

pLV3-U6-*WASL* (human)-shRNA-EGFP-Puro and pLV3-U6-MYO1B(human)-shRNA-EGFP-Puro plasmids were obtained from MiaoLingBio, China. si-h-GCM1 was obtained from RIBOBIO, China. We performed the plasmid and siRNA transfection using Lipofectamine 3000 reagent based on the manufacturer’s instructions.

### 2.8 Cell immunofluorescence

The BeWo cells were seeded in glass bottom dishes and fixed with 4% paraformaldehyde for 20 min. This was followed by permeabilization with 0.5% Triton X-100 in PBS for 2 min and blocked with 5% bovine serum albumin (BSA) for 30 min at room temperature. The primary antibody incubation, including E-cadherin Polyclonal antibody (1:50) and Beta Catenin Polyclonal antibody (1:50), occurred overnight at 4 °C, followed by a secondary antibody incubation at room temperature for 1 h in the dark. The cells were stained with 4′,6-Diamidino-2-Phenylindole (DAPI) for 3–5 min, washed, and prepared for imaging using a fluorescence microscope.

### 2.9 Mass spectrometry analysis and Co-immunoprecipitation

For mass spectrometry analysis, 1 × 10^^7^ cells were lysed using NP-40 lysis buffer. Immunoprecipitation was performed using the Classic Magnetic Protein A/G IP/Co-IP Kit (YJ201, Epizyme, China). The IP-enriched samples were separated by polyacrylamide gel electrophoresis (PAGE) and stained with Coomassie Brilliant Blue. The gel bands were excised and sent for mass spectrometry detection. Co-Immunoprecipitation (Co-IP) assays were performed using a coimmunoprecipitation (Co-IP) kit (Bes3011, BersinBio, China) following the manufacturer’s instructions. We prepared 1 × 10^7 cells for the Co-IP experiment, and the cells were lysed in NP-40 buffer. The antibody (5 μg) was incubated overnight at 4 °C, and then protein A/G magnetic beads were added and incubated for 3 h at room temperature. The antibodies used were N-WASP antibody (sc-100964, SANTA CRUZ, United States) and Myosin Ib antibody (sc-393053, SANTA CRUZ, United States). The enriched proteins on the magnetic beads were then eluted and denatured at 100 °C for Western blot analysis.

### 2.10 Cellular fraction

To obtain cellular fractions, the NE-PER Extraction Reagents (78833, Thermo Fisher, United States) were used to isolate the nuclear and cytoplasmic fractions. The nuclear fraction was confirmed using Lamin B1 as an internal control, while GAPDH was used as an internal control for the cytoplasmic fraction.

### 2.11 Bioinformatics analysis

We analyzed two datasets, GSE75010 and GSE204835, obtained from the NCBI Gene Expression Omnibus (GEO). These datasets include placental samples from patients with PE and control groups. Data preprocessing was conducted using R and its Bioconductor packages, employing the Robust Multichip Average (RMA) method for background correction and normalization of raw probe-level data. Differential expression analysis was performed using the Limma package to calculate log2 fold change and *P*-values, with the Benjamini–Hochberg method applied to adjust for multiple comparisons and to control the false discovery rate (FDR). Gene Set Enrichment Analysis (GSEA) was used to identify changes in gene sets associated with the HALLMARK_WNT_BETA_CATENIN_SIGNALING pathway. The analysis was based on ranked gene expression, and significance was assessed using *P*-values and adjusted *P*-values. Furthermore, Pearson correlation analysis was used to evaluate the relationship between *WASL* gene expression and WNT/BETA_CATENIN pathway activity, calculating the correlation coefficient (r), *P*-values, and 95% confidence intervals. Additionally, Bayesian correlation analysis was performed using the BayesFactor package to provide Bayes factors and high-density intervals (HDI) for the posterior distribution of correlations. All statistical analyses were conducted using R, with a significance threshold set at *P* < 0.05. The correlation results and GSEA findings were visualized using the ggplot2 package, presenting the data analysis results graphically.

### 2.12 Prediction and evaluation of protein-protein interaction conformations

We downloaded the three-dimensional structures of interacting proteins from the Uniprot database, and protein-protein docking analysis was performed using the HDOCKlite v1.1 local server (Yan Y. et al., 2020). The 10 leading optimal conformational models were selected based on their docking scores. A docking score lower than −200 indicated a potential interaction between the protein complexes. A high likelihood of molecular binding was suggested when the confidence score exceeded 0.7, whereas scores between 0.5 and 0.7 indicated a possible interaction, and those below 0.5 indicated a low binding probability. The binding interfaces of the protein-protein complexes were comprehensively described and systematically analyzed using the Protein-Ligand Interaction Profiler (PLIP) platform (Adasme et al., 2021). Further interaction details were supplemented by analyzing relevant interaction specifics using the PyMOL software (Mijit et al., 2023; Huang and Zou, 2008).

### 2.13 Animal model construction and specimen collection

The animal experiment was approved by the Ethics Committee of Shengjing Hospital of China Medical University (license number: 2021PS315K). The rat model of PE was established using Nω-nitro-arginine methyl ester (L-NAME; Sigma-Aldrich) based on previous reports. Here, we briefly described the procedure, including 24 nulliparous female Sprague Dawley rats (age: 8 weeks; weight: 180–200 g) and 12 males (age: 8 weeks; weight: 280–300 g) were maintained under specific pathogen-free grade animal laboratories. The temperature set was 21 °C–24 °C, and humidity was 50%–70%, with a 12-h light-dark cycle schedule. The ratio of female to male rats was 2:1. All the animals had *ad libitum* access to food and water and were subsequently divided into six groups.

Group 1, normal pregnant rats were used as controls (n = 6);

Group 2, preeclamptic group (PE, L-NAME, 75 mg/kg/D, gavage, D4–D14, n = 6);

Group 3, normal pregnant rats receiving hydroxychloroquine (HCQ, 50 mg/kg/D, D4-D19, n = 6);

Group 4, preeclamptic group (PE + HCQ, L-NAME, 75 mg/kg/D, gavage, D4-D14) receiving hydroxychloroquine (50 mg/kg/D, D4-D19, n = 6).

Gestational day zero was defined as vaginal emboli or sperm was detected by vaginal smear after rats mating. The systolic and diastolic blood pressures (SBP and DPB) of rats were measured on days 1, 4, 7, 11, 15, and 19 of pregnancy using a non-invasive rat tail arterial blood pressure monitor (BP-98A, Soforon, Japan). The mean arterial pressure (MAP) was calculated as MAP = (SBP+2×DBP)/3. Additionally, the urine of rats was collected in a metabolic cage on days 1, 11, and 19 of pregnancy. Manipulations of urine collection and gavage were performed after blood pressure measurement. On day 20 of pregnancy, rats were anesthetized using isoflurane (350 mg/kg) and sacrificed via cardiac puncture. The placenta and fetal rats were weighed, recorded, and collected.

### 2.14 Statistical analysis

All experiments were performed with at least three independent biological replicates to ensure the reliability of the results. Graphs were generated, and statistical analyses were performed using GraphPad Prism 8 software and IBM SPSS Statistics 26 (Chicago, IL, United States), respectively. Normally distributed continuous variables are presented as mean ± standard deviation (SD), and the independent T-test was performed to assess statistical differences between the two groups for this data. For experiments involving more than two groups, one-way analysis of variance (ANOVA) was used for statistical analysis. Pearson correlation analysis was utilized in the correlation analysis. Statistical significant difference was set as P < 0.05.

## 3 Results

### 3.1 Clinical characteristics of preeclampsia and control groups

The mean arterial pressures of patients with PE were significantly higher than those of the control group, including 24-h urinary protein quantifications (*P* < 0.001). In contrast, the PE group had significantly lower neonatal birth weight than the controls (*P* < 0.01). Neither maternal age nor gestational were had statistically different between the PE and control groups (Table 1).

**TABLE 1:** Clinical characteristics of each group.

Variable	Preeclampsia (n = 30)	Control group (n = 35)
Age (Years)	30.75 ± 6.01	29.81 ± 4.68
Gestational week (weeks)	32.64 ± 3.43	33.84 ± 3.12
Mean arterial pressure (mmHg)	114.40 ± 11.59^**^	94.92 ± 10.89
24-h urinary protein quantification (g/24 h)	5.76 ± 3.20^**^	0.17 ± 0.22
Neonatal birth weight (g)	1734 ± 806.9^*^	2297 ± 718.4

*P < 0.01, **P < 0.001.

### 3.2 The expression of N-WASP was downregulated in human placentas of preeclampsia

**FIGURE 1:**
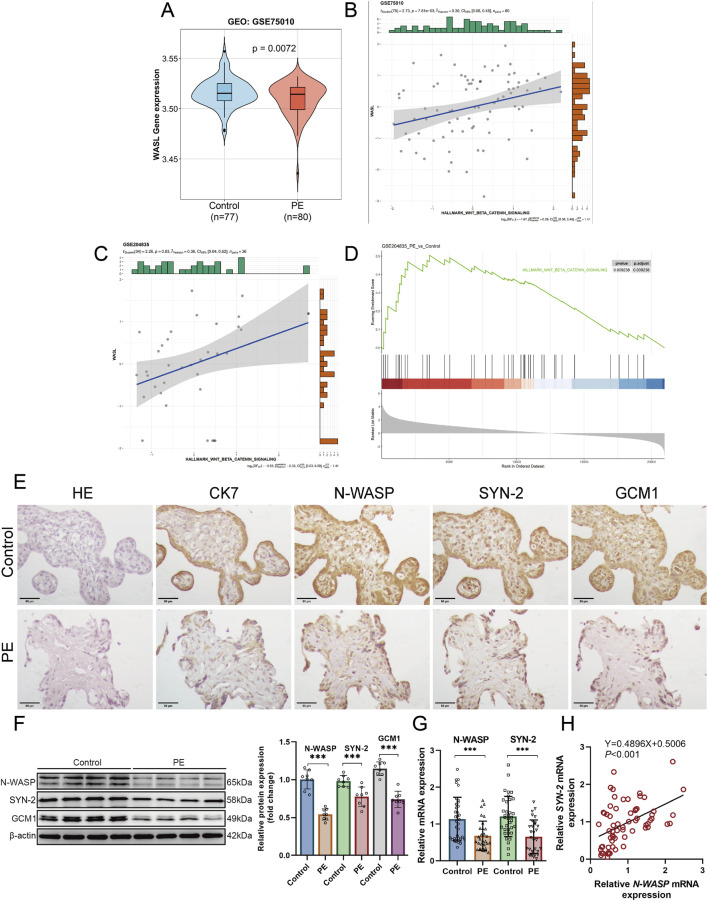
Reduced Expression of N-WASP and Syncytin-2 and Analysis of WNT/β-Catenin Pathway and WASL Gene Expression in Preeclamptic Placentas. **(A)** The violin plot depicts WASP like actin nucleation promoting factor (WASL) gene expression in the GSE75010 dataset, comparing control (n = 77) and preeclampsia (n = 80) placentas. A significant difference is observed (*P* = 0.0072). **(B)** Positive correlation between WNT/β-catenin pathway activity and WASL gene expression in GSE75010 (r = 0.36, *P* = 0.03). Histograms show expression distributions. **(C)** WASL expression positively correlated with WNT/β-catenin signaling (Pearson r = 0.36, *P* = 0.03), with moderate Bayesian support (*log* BF = -0.83) in GSE204835. **(D)** GSEA for WNT/β-catenin pathway in GSE204835 reveals significant enrichment in preeclampsia (*P* = 0.02938, adj. P = 0.03938). **(E)** Hematoxylin staining and representative immunostaining of N-WASP, syncytin-2, GCM1, and CK7 protein in the control group and preeclamptic placenta. Scale bar, 50 μm. **(F)** Representative N-WASP, syncytin-2 and the GCM1 immunoblot of preeclamptic and placenta, normalized by β-actin. **(G)** WASL (N-WASP) and syncytin-2 mRNA expression levels were quantified using quantitative polymerase chain reaction (qPCR) in placental samples from the preeclampsia group (n = 30) and control group (n = 35). The analysis revealed a significant reduction in both WASL and syncytin-2 mRNA expression levels in the preeclampsia group compared with the control group. **(H)** WASL (N-WASP) mRNA expression is positively correlated with syncytin-2 mRNA expression. SYN-2, syncytin-2; ACTB, β-actin; CK7, Cytokeratin 7; PE, preeclampsia; ***, *P* < 0.001.

The relationship between *WASL* gene expression and PE was evaluated using the GSE75010 dataset. The results showed that *WASL* gene expression was significantly lower in patients with PE than non-PE controls (*P* = 0.0072), as illustrated in the violin plot (Figure 1A). Cytokeratin 7 (CK7) was selected as a marker of CTs due to its strong positive staining in CTs (Pastuschek et al., 2021). HE staining and CK7 immunohistochemistry revealed a thinned and partially uneven trophoblast layer (Figure 1E). Notably, Western blotting assays revealed a significant decrease in the protein expressions of N-WASP, GCM1, and syncytin-2 in PE placentas compared with those of the control groups (*P* < 0.05, Figure 1F). Additionally, qPCR assays revealed that the mRNA levels of N-WASP in PE placentas (n = 30) were significantly lower than those in the control group (n = 35) (*P* < 0.05, Figure 1G). Furthermore, the Pearson correlation analysis indicated a positive correlation between N-WASP and syncytin-2 mRNA expressions (r = 0.47, 95% CI 0.26–0.64, *P* < 0.001, Figure 1H).

The significant association between N-WASP expression and activation of the WNT/β-catenin signaling pathway in the GSE75010 (n = 80) and GSE204835 datasets (n = 36) were further highlighted using the GSEA enrichment analysis results. In the GSE204835 dataset, the WNT/β-catenin signaling pathway significantly differed between the PE and control groups in enrichment analysis (*P* < 0.001) (Figure 1D). Similarly, in both the GSE75010 and GSE204835 datasets, WASL gene expression revealed a positive linear relationship between the PE and control groups (Figure 1B, C). The analyses demonstrated a significant positive correlation between WASL expression and WNT/β-catenin signaling activity in normal placental samples, suggesting a potential interaction or co-regulation under physiological conditions, rather than in the pathological context of PE.

### 3.3 Regulatory role of N-WASP in forskolin-induced BeWo cell fusion

The BeWo cell line is a human choriocarcinoma cell line that is used as an *in vitro* model for studying trophoblast differentiation due to its trophoblast-like phenotype (Wong et al., 2020; Penugurti et al., 2025). Forskolin (FSK) treatment can stimulate the fusion of BeWo cells. Hence, we investigated the expression of N-WASP protein by treating BeWo cells with different concentrations of FSK (0, 10, 25, 50 μM). We measured the expression level of β-HCG mRNA, a marker of trophoblast cell fusion, after 48 h of FSK treatment. Notably, HCG is a crucial hormonal indicator of trophoblast differentiation. Simultaneously, we assessed the expression of E-cadherin (E-cad) protein, a key marker distinguishing cytotrophoblasts (CTs) and syncytiotrophoblasts (STs) (Wang et al., 2024). Additionally, we quantified the cell fusion index as an indicator of trophoblast cell fusion.

**FIGURE 2:**
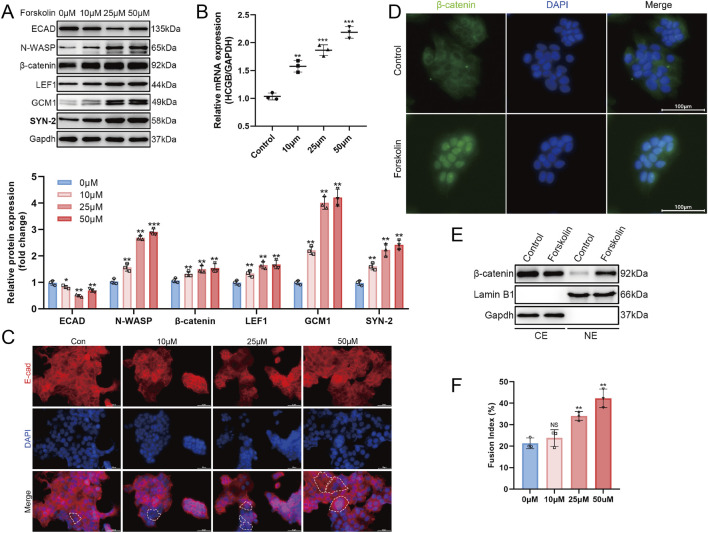
N-WASP protein expression during trophoblast cell syncytialization. **(A)** Western blot showed the expression of E-cadherin (ECAD), neural Wiskott‐Aldrich syndrome protein (N-WASP), β-catenin, LEF1, GCM1, and syncytin-2 (SYN2) protein in BeWo cells stimulated by forskolin (FSK) gradient concentration (0 μm, 10 μm, 25 μm, and 50 μm). Data were normalized to GADPH. **(B)** Quantitative real-time polymerase chain reaction (RT-qPCR) result showed the expression of β-hCG mRNA in BeWo cells stimulated by FSK gradient concentration. Data were normalized to GADPH. **(C)** Immunofluorescence staining of E-cadherin (red) and nuclei (DAPI, blue) showing changes in E-cadherin localization and expression in cells treated with gradient concentration of FSK. Dashed lines highlight cellular boundaries. Scale bar = 50 μm. **(D)** Immunofluorescence images of β-catenin (green) and nuclei (DAPI, blue) in 0μm (Control) and 25 μm forskolin-treated Bewo cells. FSK treatment results in increased nuclear β-catenin. Scale bar = 100 μm. **(E)** Western blot analysis of β-catenin in cytoplasmic (CE) and nuclear (NE) extracts from control and 25 μm FSK-treated cells, with Lamin B1 and GAPDH as nuclear and cytoplasmic markers, respectively. **(F)** Fusion index of cells treated with varying concentrations of FSK. **, *P* <0.01, ***, *P* < 0.001, NS = not significant.

The BeWo cells showed significant fusion characteristics after FSK treatment. Moreover, qPCR assays revealed a significant increase in β-HCG mRNA expression with higher FSK concentrations, confirming the promotion of fusion (Figure 2B). Meanwhile, Western blotting assays revealed a significant decrease in E-cadherin expression levels with increasing FSK concentrations (Figure 2A). Immunofluorescence analysis further showed a marked reduction in E-cadherin distribution on the cell membrane, accompanied by the appearance of multinucleated cells, characteristic of cell fusion (Figure 2C). The fusion index results provided additional support, revealing a significant increase in the fusion level of BeWo cells with higher FSK concentrations (Figure 2F, *P* < 0.01). During the FSK-induced BeWo cell fusion, Western blotting assays revealed that the expression levels of N-WASP, GCM1, and syncytin-2 (SYN-2) proteins were significantly upregulated and peaked at 25 μM; however, it plateaued at higher concentrations (50 μM) (Figure 2A). Additionally, the Wnt signaling pathway was activated during this process. This was confirmed by the nuclear accumulation of β-catenin, confirmed through Western blotting analysis of cytoplasmic and nuclear extracts, where β-catenin levels in the nucleus increased significantly after FSK treatment (Figure 2E). In addition, immunofluorescence analysis revealed an enhanced nuclear β-catenin signal intensity after FSK treatment at 25 μM, providing visual confirmation of β-catenin nuclear translocation (Figure 2D). Furthermore, the expression level of LEF1, a downstream transcription factor of the Wnt signaling pathway, was significantly increased with higher FSK concentrations than with FSK at 0 μM, which was confirmed by Western blotting assays (Figure 2A).

**FIGURE 3:**
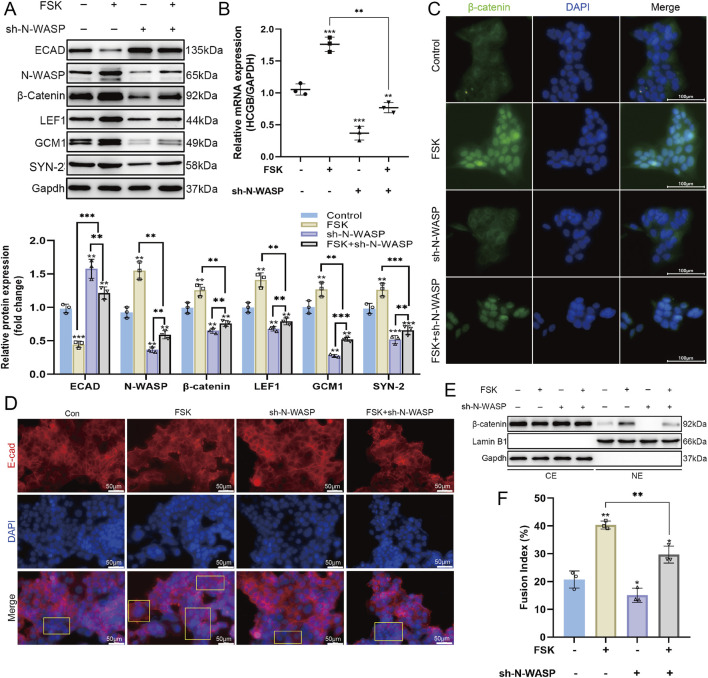
Role of N-WASP in the forskolin-stimulated BeWo cells. **(A)** During the process of forskolin (FSK)-induced BeWo cell fusion, western blot showed the expression of neural Wiskott-Aldrich syndrome protein (N-WASP) was upregulated, and the expression levels of β-catenin and LEF1 proteins also increased, indicating the activation of the Wnt/β-catenin signaling pathway. Additionally, the expression levels of syncytin-2 and glial cells missing 1 (GCM1) were significantly elevated. **(B)** Quantitative polymerase chain reaction (qPCR) analysis revealed that β-HCG mRNA expression, normalized to GAPDH, across the same treatment groups, highlighting the impact of N-WASP knockdown on β-HCG transcription during FSK-induced BeWo cell fusion. **(C)** Immunofluorescence images of β-catenin (green) and nuclei (DAPI, blue) across treatments. FSK treatment alone increases the nuclear localization of β-catenin, which is reduced when N-WASP is knocked down (FSK + sh-N-WASP). Scale bar = 100 μm. **(D)** Immunofluorescence staining of E-cadherin (red) and nuclei (DAPI, blue) illustrating changes in E-cadherin expression and cellular boundary morphology across treatments. FSK enhances E-cadherin localization along cell-cell contacts, while sh-N-WASP alters this pattern. Scale bar = 50 μm. **(E)** Western blot of β-catenin in cytoplasmic (CE) and nuclear (NE) extracts under the same treatment conditions, with Lamin B1 and GAPDH as nuclear and cytoplasmic markers, respectively. FSK promotes β-catenin nuclear translocation, which is diminished by N-WASP knockdown. **(F)** Fusion index quantifying cell fusion percentage across treatments. FSK significantly increases cell fusion, an effect partially reduced by N-WASP knockdown. ECAD, E-cadherin; SYN-2, syncytin-2; ACTB, β-actin. *, *P* < 0.05, **, *P* < 0.01, ***, *P* < 0.001.

We established stable N-WASP knockdown BeWo cells using lentiviral shRNA targeting N-WASP and treated with FSK to induce syncytialization to evaluate the role of N-WASP in BeWo cell fusion. Compared with the group treated with FSK alone, N-WASP knockdown significantly inhibited the FSK-induced upregulation of β-HCG mRNA expression, confirmed by qPCR assays (Figure 3B). Furthermore, fusion index analysis revealed that the increase in fusion levels was substantially impaired after N-WASP knockdown (Figure 3F). Additionally, Western blotting assays showed that the expression level of E-cadherin was significantly elevated following N-WASP knockdown (Figure 3A), and immunofluorescence analysis revealed increased E-cadherin distribution on the cell membrane (Figure 3D). The Western blot results further showed that after N-WASP knockdown, the FSK-induced expression levels of syncytin-2 and GCM1 proteins were significantly reduced (Figure 3A). Moreover, N-WASP knockdown significantly suppressed the expression of β-catenin and LEF1, as revealed using the Western blotting assays (Figure 3A), and decreased the nuclear distribution of β-catenin (Figure 3C, E). This suggests that the activation of the Wnt/β-catenin signaling pathway was impaired. These findings collectively demonstrate that N-WASP knockdown significantly inhibits FSK-induced BeWo cell fusion, reduces the expression levels of syncytin-2 and GCM1 proteins, and suppresses the activation of the Wnt/β-catenin signaling pathway.

### 3.4 Inhibition of the β-catenin pathway counteracts N-WASP-mediated activation of GCM1 and Syncytin-2 expression in trophoblast cell fusion

**FIGURE 4:**
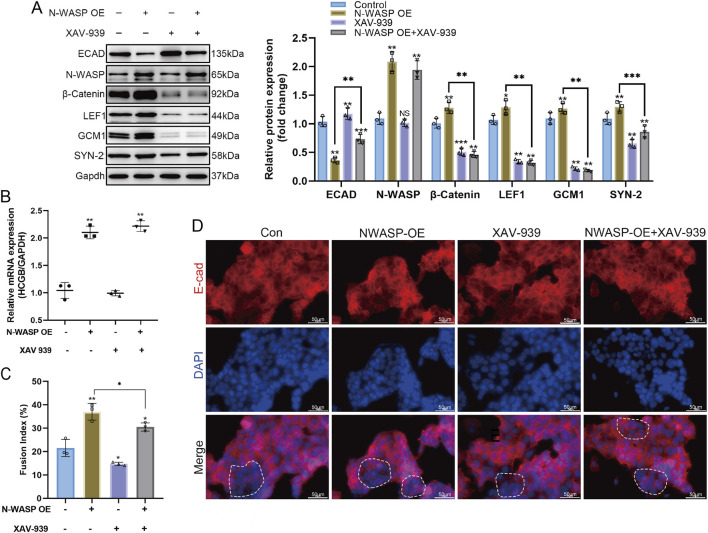
The fusion-promoting effect of N-WASP Overexpression in BeWo Cells is significantly attenuated by the Wnt/β-catenin pathway inhibitor XAV-939. **(A)** Western blot analysis of protein levels in BeWo cells under different treatment conditions. neural Wiskott-Aldrich syndrome protein (N-WASP) overexpression significantly increased the expression of fusion-related markers, while XAV-939 treatment counteracted this effect. **(B)** N-WASP overexpression led to a significant increase in β- human chorionic gonadotropin (HCG) mRNA levels, which was reduced upon co-treatment with XAV-939. **(C)** Quantification of fusion index (%) is shown in the adjacent bar graph. Forskolin-induced fusion was enhanced by N-WASP overexpression; however, XAV-939 treatment partially reversed this effect. **(D)** Immunofluorescence staining for E-cadherin (E-cad; red) and DAPI nuclear stain (blue) in BeWo cells across different conditions. Merged images indicate cell-cell adhesion structures and cellular fusion events. In the Control and N-WASP overexpression (OE) groups, E-cadherin staining reveals diminished continuity of E-cadherin staining along cell borders, with prominent fusion in the N-WASP OE group. XAV-939 treatment (both alone and with N-WASP OE) leads to reduced cell fusion, as evidenced by distinct cell-cell borders of E-cadherin staining along cell borders. Scale bars represent 50 μm. **, *P*<0.01, ***, *P*<0.001.

We established BeWo cells with stable overexpression of N-WASP and inhibited the β-catenin signaling pathway using XAV-939, a WNT pathway inhibitor, to investigate the role of N-WASP in forskolin-induced BeWo cell fusion and its relationship with the WNT/β-catenin signaling pathway. The results showed that N-WASP overexpression significantly promoted BeWo cell fusion, confirmed by a substantial increase in β-HCG mRNA levels in the qPCR assays (Figure 4B) and fusion index (Figure 4C). The Western blot analysis showed that N-WASP overexpression resulted in a significant decrease in E-cadherin protein levels, whereas the expression levels of β-catenin, GCM1, and SYN-2 were significantly elevated (Figure 4A). In addition, immunofluorescence staining further confirmed these findings, showing that N-WASP overexpression weakened E-cadherin fluorescence signals, indicating reduced cell-cell junction integrity and enhanced cell fusion (Figure 4D). However, N-WASP protein expression levels remained unchanged when XAV-939 was added, as shown by Western blotting assays, nonetheless, cell fusion was notably inhibited. This inhibition was reflected by a significant decrease in β-HCG mRNA levels (Figure 4B) and fusion index (Figure 4C), along with an increase in E-cadherin protein expression via Western blotting assays (Figure 4A). Additionally, stronger fluorescence signals were observed in the Immunofluorescence staining (Figure 4D). Moreover, Western blot assays showed that the protein expression levels of β-catenin and LEF1, key regulators of the WNT/β-catenin signaling pathway and trophoblast fusion, were significantly reduced upon XAV-939 treatment (Figure 4A). These findings indicate that N-WASP overexpression promotes BeWo cell fusion by upregulating β-catenin, GCM1, and SYN-2 expression. Conversely, the WNT/β-catenin signaling pathway inhibitor XAV-939 effectively suppresses this effect, suggesting that N-WASP regulates BeWo cell fusion by activating the WNT/β-catenin signaling pathway.

### 3.5 Interaction of N-WASP and MYO1B activates FAK/β-catenin signaling pathway to promote trophoblast cell fusion

**FIGURE 5:**
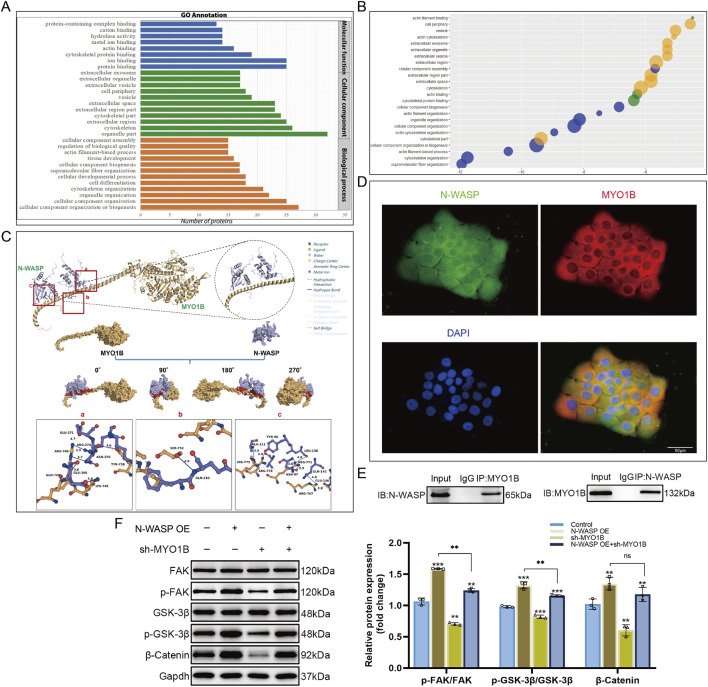
Interaction between N-WASP and MYO1B activates the FAK/β-catenin signaling pathway. **(A)** Identification and analysis of neural Wiskott-Aldrich syndrome protein (N-WASP) interacting proteins in N-WASP overexpressing BeWo cell models using co-immunoprecipitation coupled with mass spectrometry. Gene ontology (GO) enrichment analysis results are presented as a bar chart, showing that the enriched functions are primarily associated with protein binding, cytoskeletal organization, and cellular component biogenesis. **(B)** Functional enrichment bubble plot of myosin 1B (MYO1B), displaying various biological interaction networks associated with cell fusion. Each bubble represents a significantly enriched GO term, with the bubble size and color indicating the level of enrichment. **(C)** Molecular docking analysis of MYO1B and N-WASP binding using the HDOCK server, with the binding interface visualized in surface representation. MYO1B is shown in yellow, N-WASP in purple, and the protein-protein contact area in red. Interface interactions analyzed by Protein-Ligand Interaction Profiler (PLIP) are as follows: **(A)** A salt bridge is formed between ARG-746 of MYO1B and GLU-271 of N-WASP (yellow dashed line), along with hydrogen bonds between ARG-746 and N-WASP residues ARG-274 and ASN-270 (blue solid lines). Additional hydrogen bonds are observed between GLN-749 and LYS-745 of MYO1B and GLU-266 of N-WASP, as well as between TYR-738 of MYO1B and ASN-270 of N-WASP. **(B)** A hydrogen bond is formed between SER-752 of MYO1B and GLN-210 of N-WASP. **(C)** Hydrogen bonds are observed between ALA-111 of N-WASP and HIS-775 of MYO1B, ARG-778 of MYO1B and TYR-95 of N-WASP, ARG-711 of MYO1B with ASN-97 and LEU-138 of N-WASP, and ARG-767 of MYO1B with GLN-142 and GLU-146 of N-WASP, forming additional hydrogen bonds and salt bridges. Gray dashed lines indicate regions of multiple hydrophobic interactions. **(D)** Immunofluorescence staining shows colocalization of N-WASP (green) and MYO1B (red) within cells. DAPI staining marks the cell nuclei (blue). The merged image indicates colocalization of N-WASP and MYO1B, suggesting a possible interaction between them. Scale bars represent 50 μm. **(E)** Co-immunoprecipitation experiment verifies the interaction between N-WASP and MYO1B in BeWo cells. The left panel shows the detection of N-WASP after MYO1B immunoprecipitation (IP), and the right panel shows the detection of MYO1B after N-WASP immunoprecipitation, confirming a physical interaction between the two proteins in cells. *(F)* Western blot analysis of the effects of N-WASP overexpression and MYO1B knockdown on the FAK/β-catenin signaling pathway. Results indicate significant changes in the phosphorylation levels of FAK (p-FAK/FAK), GSK-3β (p-GSK-3β/GSK-3β), and β-catenin expression following MYO1B knockdown. **, *P*<0.01, ***, *P*<0.001.

We constructed an N-WASP-overexpressing BeWo cell model and used co-immunoprecipitation and proteomic mass spectrometry to identify proteins interacting with N-WASP to clarify how N-WASP activates the β-catenin signaling pathway and promote BeWo cell fusion. The results showed that 88 encoding proteins were identified, of which 15 were actin-related proteins (Figure 5A). Gene ontology (GO) analysis further revealed that myosin 1B (MYO1B) is closely associated with biological processes such as cell morphology changes, movement, and adhesion, suggesting that it may contribute substantially to cell fusion (Figure 5B). Subsequently, we obtained the predicted full-length structures of N-WASP and MYO1B from the UniProt database using AlphaFold and performed protein-protein docking using HDOCK. The optimal docking model showed high binding affinity (docking score for model 1: 302.45, confidence level: 0.9547), indicating a high likelihood of interaction. PLIP analysis further revealed the interaction details between MYO1B and N-WASP at the protein surface (Figure 5C). The binding free energy calculated by the HawkDock server was −40.33 kcal/mol. In addition, Co-immunoprecipitation and immunofluorescence assays confirmed the cytoplasmic colocalization of N-WASP and MYO1B (Figure 5D-E).

Through literature research, we found that MYO1B is associated with the development of various malignancies and enhances tumor cell migration and invasion in colorectal cancer by activating the RhoA/ROCK/FAK signaling pathway (Chen et al., 2022). In mouse osteoclasts, FAK activation stabilizes β-catenin and promotes its nuclear translocation (Sun et al., 2016). We transfected MYO1B-shRNA into N-WASP-overexpressing BeWo cells to explore how N-WASP influences the FAK signaling pathway via MYO1B. The Western blot results showed that, compared with the N-WASP overexpression group, MYO1B knockdown significantly increased the p-FAK/FAK ratio (0.97 ± 0.05 vs. 1.24 ± 0.02, *P* < 0.001), whereas p-GSK-3β/GSK-3β and β-catenin levels increased; however, to a lesser extent than in the N-WASP overexpression group (1.00 ± 0.01 vs. 1.42 ± 0.08, *P* < 0.001) (Figure 5F).

**FIGURE 6:**
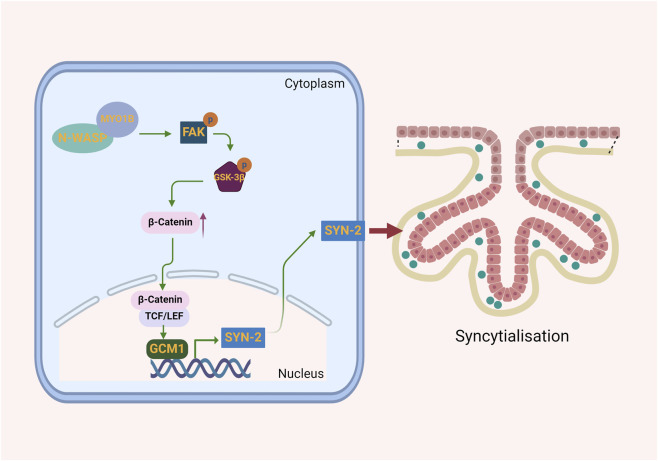
Illustration of how N-WASP promotes syncytin-2 expression to drive trophoblast syncytialization. N-WASP, through its interaction with MYO1B, activates the FAK signaling pathway, stabilizing β-catenin and promoting its nuclear translocation. In the nucleus, β-catenin enhances GCM1 expression, which drives syncytin-2 transcription. Syncytin-2 facilitates trophoblast fusion, forming the syncytial layer essential for syncytialization.

*In vitro*, N-WASP was shown to interact with MYO1B, leading to the activation of the FAK signaling pathway. This activation stabilizes β-catenin and promotes its nuclear translocation, where it upregulates GCM1, a key transcription factor driving syncytin-2 expression (Figure 6).

### 3.6 Expression of N-WASP, GCM1 and Syncytin-2 in preeclampsia rat model

**FIGURE 7:**
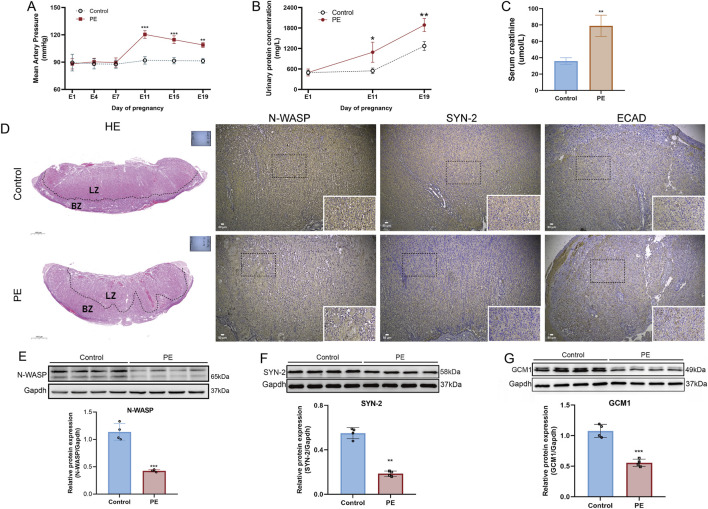
Establishment of preeclampsia rat model and expression changes of N-WASP, GCM1, and syncytin-2 in placental tissue. **(A)** Mean arterial pressure (MAP) is significantly elevated in the preeclampsia (PE) group from day 7 of pregnancy, confirming the development of hypertension characteristic of PE. **(B)** Urinary protein levels increase substantially in the PE group from day 11 of pregnancy. **(C)** Elevated serum creatinine levels in the PE group suggest renal dysfunction. **(D)** Histological and immunohistochemical analysis reveals disrupted placental architecture in the PE group, with reduced labyrinth zone (LZ) and decreased expression of neural Wiskott-Aldrich syndrome protein (N-WASP), syncytin-2 (SYN-2), and elevated expression of E-cadherin (ECAD). **(E)** Western blot results show significantly reduced N-WASP expression in the PE group. HE staining scale bar=100 μm, immunohistochemistry scale bar=50 μm. **(F)** Decreased syncytin-2 levels in the PE group. **(G)** GCM1 expression is significantly lower in the PE group.

On gestational day 19, rats with PE showed significantly elevated mean arterial pressure (MAP) and urine protein levels compared with normal pregnant rats, indicating the successful establishment of the PE model (Figure 7A-C). Furthermore, the visual observation of HE staining revealed that placental structures in the control group were clear, with abundant vasculature between the placental villi. The villous cells showed normal distribution and morphology, with an intact labyrinth zone structure. In contrast, PE rats exhibited a significantly thinned basal zone and a reduced number of trophoblast cells in the labyrinth zone (Figure 7D). Regarding protein expression, the levels of N-WASP, GCM1, and syncytin-2 in the PE group were significantly lower than those in normal pregnant rats, whereas E-cadherin protein immunohistochemical staining was higher in the PE group than in the control group (Figure 7E-F).

**FIGURE 8:**
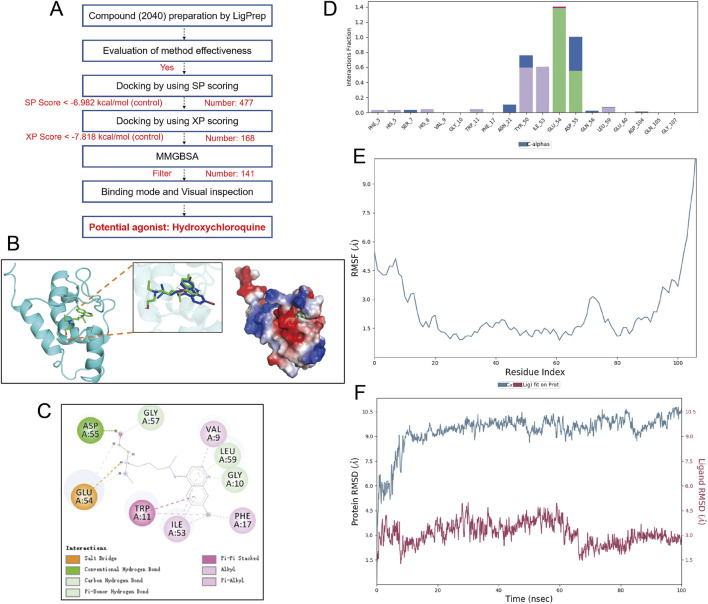
Screening, binding mode, and molecular dynamics simulation analysis of Hydroxychloroquine as a potential agonist for N-WASP protein. **(A)** virtual screening workflow for the neural Wiskott-Aldrich syndrome protein (N-WASP) target. 2040 compounds were prepared using LigPrep and subjected to multiple screening steps (SP scoring, XP scoring, and MMGBSA analysis), ultimately identifying hydroxychloroquine (HCQ) as a potential agonist. **(B)** Binding mode of N-WASP protein with HCQ. The left panel shows the three-dimensional structure of the N-WASP-HCQ complex, the middle panel displays the electrostatic surface of the protein, and the right panel shows the superimposition of HCQ with the control molecule WSK. This structural analysis illustrates the binding pocket location and relative conformation of HCQ within the N-WASP target. **(C)** Detailed binding mode of HCQ with N-WASP protein. The figure shows key interactions of HCQ within the N-WASP active site, including strong hydrogen bonds with GLU-54 and ASP-55 residues. These hydrogen bonds are maintained for over 60% of the time during molecular dynamics simulation, indicating a sustained and effective interaction of HCQ at the protein binding site. These hydrogen bonds contribute significantly to the stability of HCQ within the protein pocket. **(D)** Interaction fraction of HCQ with N-WASP protein. Key residues involved in HCQ binding to N-WASP are shown with their interaction types and frequencies, particularly the hydrogen bond interactions with GLU-54 and ASP-55, further supporting the stability of the molecule at the binding site. **(E)** Root Mean Square Fluctuation (RMSF) plot of the HCQ-N-WASP complex. RMSF represents the conformational fluctuations of each amino acid residue during the simulation. Peaks indicate regions with high residue fluctuations, mainly concentrated in the protein's hinge region, which has inherent flexibility and thus undergoes conformational changes during the simulation. Lower RMSF values indicate that HCQ binding helps stabilize the protein. **(F)** Root Mean Square Deviation (RMSD) plot of the N-WASP protein-HCQ complex. The average RMSD of the complex is less than 10 Å and achieves dynamic equilibrium within 20 ns, indicating that HCQ matches well with the N-WASP target and can form a stable complex.

During the virtual screening for N-WASP agonists, 141 compounds with the highest scores were selected from a pool of 2,040. Re-docking with the known natural ligand WSK and comparison with the co-crystal structure yielded a root mean square deviation of <0.5 Å, verifying the reliability of the screening method. Based on the screening results, hydroxychloroquine was selected for visualization analysis, revealing good binding and high affinity with the N-WASP target protein (binding energy: 9.248 kcal/mol; MMGBSA score: 84.71 kcal/mol) (Figure 8).

**FIGURE 9:**
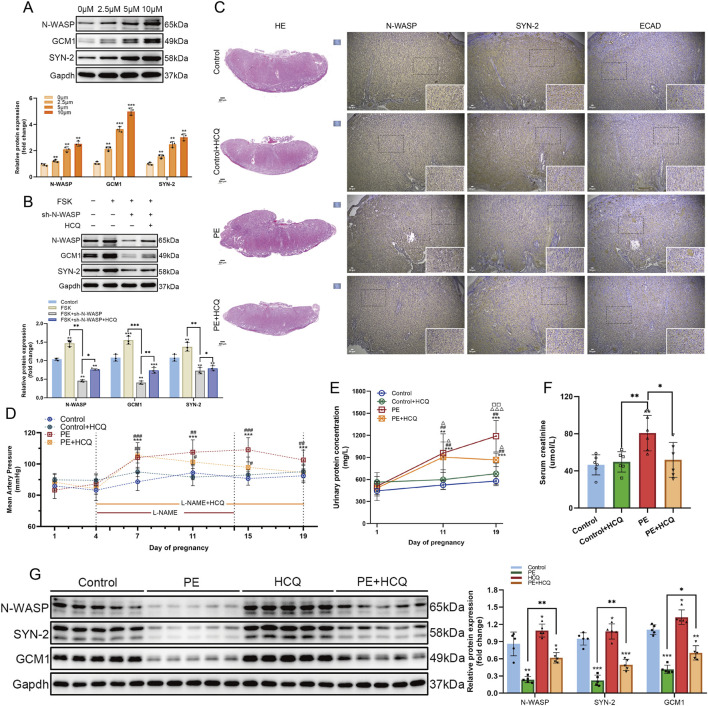
Effects of N-WASP expression changes in a rat model of preeclampsia. **(A)** Western blot analysis of neural Wiskott-Aldrich syndrome protein (N-WASP), glial cells missing 1 (GCM1), and syncytin-2 (SYN-2) protein expression in BeWo cells treated with different concentrations of hydroxychloroquine (HCQ) (0, 2.5, 5, and 10 μM). The results indicate that HCQ upregulates N-WASP expression in a dose-dependent manner, with a concomitant significant increase in GCM1 and SYN-2 expression. **, *P* < 0.01, ***, *P* < 0.001, compared with the 0 μM group. **(B)** In a forskolin (FSK)-induced BeWo cell model, Western blot results show that N-WASP-shRNA significantly reduced N-WASP expression. However, HCQ treatment reversed this downregulation and concurrently upregulated GCM1 and SYN-2 expression. *, *P* < 0.05, **, *P* < 0.01, ***, *P* < 0.001. **(C)** Immunohistochemical analysis of N-WASP, SYN-2, and E-cadherin (E-CAD) expression in placental tissues from the control, control+HCQ, preeclampsia (PE), and PE+HCQ groups. Results reveal decreased N-WASP and SYN-2 expression and elevated E-CAD expression in the PE group. HCQ treatment restored N-WASP and SYN-2 levels while reducing E-CAD expression. **(D)** Mean arterial pressure (MAP) changes during pregnancy in each group. MAP was significantly elevated in the PE group, whereas HCQ treatment markedly reduced MAP levels in PE rats (#*P* < 0.05, ##*P* < 0.01 compared with the PE group)., compared with day 1 of pregnancy, **, *P* < 0.001; #, compared with the Control group at the same gestational day, #, *P* < 0.05, ##, *P* < 0.01, ###, *P* < 0.001; △, compared with the Control+HCQ group at the same gestational day; □, compared with the PE group at the same gestational day. **(E)** Changes in urinary protein concentration during pregnancy across groups. Urinary protein levels were significantly increased in the PE group, while HCQ treatment markedly reduced urinary protein levels (△*P* < 0.05, △△*P* < 0.01 compared with the PE group). , compared with day 1 of pregnancy, *, *P* < 0.01, ***, *P* < 0.001; #, compared with the Control group at the same gestational day, ##, *P* < 0.01; △, compared with the Control+HCQ group at the same gestational day, △, *P* < 0.05, △△△, *P* < 0.001; □, compared with the PE group at the same gestational day, □□, *P* < 0.01. **(F)** Comparison of serum creatinine levels among groups. Serum creatinine levels were significantly elevated in the PE group but were substantially decreased following HCQ treatment. *, *P* < 0.05, **, *P* < 0.01, ***, *P* < 0.001. **(G)** Western blot analysis shows downregulation of N-WASP, GCM1, and SYN-2 expression in the PE group. HCQ treatment effectively restored N-WASP expression and simultaneously upregulated GCM1 and SYN-2 levels. *, *P* < 0.05, **, *P* < 0.01, ***, *P* < 0.001.

During the FSK-induced BeWo cells, we added gradient concentrations of HCQ and observed a corresponding increase in the expression levels of N-WASP, GCM1, and syncytin-2 with increasing HCQ concentration. Additionally, in FSK-treated shRNA-N-WASP BeWo cells, the expression levels of N-WASP, GCM1, and syncytin-2 were initially reduced; however, this downward trend in protein expression was reversed after adding HCQ (Figure 9A-B). We further validated our hypothesis using a PE rat model. Notably, on day 19 of gestation, preeclamptic rats treated with HCQ showed a significant decrease in MAP and serum creatinine levels compared with the untreated PE rats. Additionally, the urine protein concentration was reduced in the HCQ + PE group; however, it remained elevated relative to the control and Control + HCQ groups (Figure 9D-F). Furthermore, histological evaluation through HE staining revealed enhanced placental architecture in HCQ-treated PE rats, characterized by increased clarity and a denser vascular network within the placental villi. Nevertheless, the permeability of trophoblast folds in the labyrinth zone showed only partial improvement (Figure 9C). Moreover, HCQ administration was associated with a marked upregulation of N-WASP protein expression compared with the untreated PE rats, accompanied by a significant increase in GCM1 and syncytin-2 levels (Figure 9G). While our study demonstrates that HCQ treatment leads to an upregulation of N-WASP expression and ameliorates symptoms in the PE rat model, as a small molecule compound, HCQ may have multiple targets and biological effects. Our findings suggest that increased N-WASP expression may alleviate PE symptoms by promoting syncytin-2 levels and enhancing trophoblast fusion.

## 4 Discussion

In this study, we systematically explored, for the first time, the molecular mechanisms of N-WASP in regulating trophoblast syncytialization and its role in the pathogenesis of PE. The findings reveal that N-WASP expression is significantly reduced in PE placentas. Further analysis shows that N-WASP interacts with MYO1B to activate the FAK and β-catenin signaling pathways, thereby upregulating the transcription factor GCM1, which promotes syncytin-2 expression and enhances trophoblast fusion. These discoveries provide novel insights into the molecular pathogenesis of PE and lay a theoretical foundation for developing diagnostic and therapeutic strategies.

The pathogenesis of PE is closely associated with abnormal placental development and dysfunction (Langbein et al., 2008; Choi et al., 2022). Normal placental function depends on the differentiation and fusion of trophoblasts, particularly the transition of CTs into STs (Xiao et al., 2020). This process, which is critical for placental barrier formation, relies on the coordinated expression of multiple proteins, including fusogens for instance, syncytinsand their receptors (Roberts et al., 2021), and the dynamic remodeling of the cytoskeleton, particularly actin filaments (Ishikawa et al., 2014). Notably, Syncytin-2, a functional fusogenic protein, is essential for maintaining placental structure and function. Its dysregulated expression directly impairs trophoblast syncytialization and is closely associated with the occurrence of PE (Vargas et al., 2011; Chen et al., 2008).

N-WASP critically regulates the actin cytoskeleton network, with its C-terminal VCA domain interacting with the Arp2/3 complex to control actin polymerization and the formation of filamentous actin (Yan P. et al., 2020). In this study, N-WASP expression was significantly downregulated in preeclamptic placental tissues. Considering its role in myoblast fusion (Mukherjee et al., 2011; Gruenbaum-Cohen et al., 2012), we hypothesized that reduced N-WASP expression may significantly impact placental structure and function. Additionally, N-WASP mRNA levels positively correlated with syncytin-2 expression, suggesting that N-WASP may influence trophoblast fusion by regulating syncytin-2 expression. These findings highlight the crucial role of N-WASP in trophoblast syncytialization and provide new directions for investigating the pathogenesis of PE. Reportedly, dynamic cytoskeletal remodeling is critical for trophoblast syncytialization and PE development. For example, Wang et al. reported that reduced α-tubulin detyrosination levels in preeclamptic placentas lead to microtubule depolymerization, which results in decreased syncytin-2 aggregation on the cell membrane and inhibits trophoblast fusion (Wang R. et al., 2019). Furthermore, the Par6 protein, which is highly expressed in preeclamptic placentas, negatively regulates trophoblast syncytialization by modulating tight junctions and cytoskeletal dynamics (Sivasubramaniyam et al., 2013).

Additionally, bioinformatics analysis revealed a significant association between N-WASP expression and the activation of the Wnt/β-catenin signaling pathway in preeclamptic placentas. Wnt proteins are involved in embryonic development and organ formation, and the Wnt/β-catenin signaling pathway critically regulates trophoblast syncytialization (Liu et al., 2022; Matsuura et al., 2011). Dysfunction of several factors in this pathway impairs labyrinth zone formation and syncytiotrophoblast development in mice (Kaloglu et al., 2020). *In vitro,* experiments reveal that N-WASP initiates the activation of the Wnt/β-catenin signaling pathway during forskolin-induced BeWo cell fusion, which upregulates GCM1 expression and subsequently regulates syncytin-2 levels. This result aligns with previous studies indicating that N-WASP positively regulates the Wnt/β-catenin signaling pathway in epithelial tissues, where it substantially contributes to cell adhesion and signal transduction during mouse hair follicle cycling (Lyubimova et al., 2010). Additionally, N-WASP overexpression in cervical cancer was associated with the Wnt/β-catenin signaling pathway (Hou et al., 2021). Studies report that GCM1 is a key downstream factor in the Wnt/β-catenin pathway, with its expression directly regulated by this signaling cascade. Specifically, β-catenin directly activates GCM1 transcription by binding to TCF sites in its second intron and promotes BeWo cell fusion by regulating the GCM1/syncytin pathway (Matsuura et al., 2011). Moreover, activation of the canonical Wnt/β-catenin pathway is sufficient to induce SynT-II cell differentiation in mouse trophoblast stem cells, which are GCM1-positive and necessary for the formation of labyrinth villous branches (Zhu et al., 2017). These findings support our experimental results, indicating that N-WASP upregulates GCM1 expression by activating the Wnt/β-catenin signaling pathway, ultimately promoting syncytin-2 expression.

We further explored how N-WASP activates the Wnt/β-catenin signaling pathway during trophoblast fusion. We identified a novel molecular mechanism involving the interaction between N-WASP and MYO1B. This interaction likely activates the FAK signaling pathway, which promotes β-catenin nuclear translocation and subsequent activation of the Wnt/β-catenin signaling pathway. Notably, MYO1B, a member of the actin superfamily, is a monomeric motor protein that binds actin to maintain plasma membrane structure and regulate actin cytoskeleton dynamics, significantly contributing to migration and motility (Pernier et al., 2019). Additionally, MYO1B has been implicated in the progression of various malignant tumors. In colorectal cancer, it enhances tumor cell migration and invasion by activating the RhoA/ROCK/FAK signaling pathway, thereby promoting metastasis (Chen et al., 2022). FAK, on the other hand, is essential for embryonic implantation and placental formation (Zhao and Guan, 2011; Shen et al., 2005). Its activation stabilizes the β-catenin protein and facilitates its nuclear translocation (Sun et al., 2016). In this study, we demonstrated that N-WASP interacts with MYO1B to activate the FAK signaling pathway, thereby promoting β-catenin nuclear translocation and regulating syncytin-2 expression, which is closely associated with trophoblast fusion.

In the PE rat model, we observed significantly reduced expression of N-WASP, GCM1, and syncytin-2, accompanied by decreased labyrinth zone area and abnormal villous tissue structure. Interventions that increased N-WASP expression significantly improved labyrinth zone development and alleviated PE symptoms in rats. These findings strongly support the critical role of N-WASP in placental development and PE pathogenesis. However, the animal models used in this study, while replicating some pathological features of PE, differed significantly from human placental structure and function. Therefore, limiting the comprehensive understanding of the complex pathogenesis of PE. Additionally, the systemic knockout of N-WASP maybe lethal to the embryos, and due to cost constraints, transgenic animal models were not used in this study, limiting further verification of causal relationships.

This study has other limitations. The use of Forskolin-induced BeWo cells as an *in vitro* model, though widely accepted, does not fully reflect the complex *in vivo* placental environment, including maternal-fetal interactions and hormonal regulation. Furthermore, the study provides a partial understanding of the molecular mechanisms underlying N-WASP function. A limitation of this study is that, while the necessity of MYO1B for the interaction with N-WASP and the activation of the FAK/β-catenin pathway has been demonstrated, the sufficiency of this interaction in pathway activation remains unverified. Additionally, the exploration of upstream regulators of N-WASP remains incomplete. Further research is needed to address these limitations.

In this study, we provided the first evidence that N-WASP expression is significantly reduced in preeclamptic placental tissues and revealed its critical role in promoting trophoblast syncytialization. The specific mechanism involves the interaction of N-WASP with MYO1B, which activates the FAK/β-catenin signaling pathway, upregulating the transcription factor GCM1 and promoting the transcription and expression of syncytin-2. Reduced N-WASP expression may inhibit trophoblast syncytialization, ultimately leading to the development of PE. These findings deepen our understanding of the pathogenesis of PE and provide an important theoretical basis and research direction for exploring diagnostic and therapeutic strategies targeting N-WASP.

## Data Availability

The original contributions presented in the study are included in the article/supplementary material, further inquiries can be directed to the corresponding authors.
